# From Basic Research to New Tools and Challenges for the Genotoxicity Testing of Nanomaterials

**DOI:** 10.3390/nano10102073

**Published:** 2020-10-20

**Authors:** Valérie Fessard, Fabrice Nesslany

**Affiliations:** 1 Unit of Toxicology of Contaminants, Fougères Laboratory, French Agency for Food, Environmental and Occupational Health & Safety, 10B rue C. Bourgelat, 35306 Fougères, France; 2 Genotoxicology Department, Institut Pasteur de Lille, 1, Rue du Professeur Calmette, 59000 Lille, France

Genotoxicity is one of the key endpoints investigated as early as possible before marketing a product. Several assays can be performed to evaluate the genotoxicity and mutagenicity of compounds, covering the detection of DNA lesions and of DNA repair activities as well as the quantification of the three types of mutations at the gene, chromosome and genome levels. Most of these tests have an established or draft guideline from the Organization for Economic Co-operation and Development (OECD). Nevertheless, if these assays have been developed for chemicals, they are not fully suitable for testing the genotoxicity/mutagenicity of nanomaterials, and the adaptation of the experimental conditions for such compounds is still debated. This Special Issue “From Basic Research to New Tools and Challenges for the Genotoxicity Testing of Nanomaterials” aimed to provide new results on a large range of nanomaterials using a broad panel of genotoxicity assays and to solve some of the numerous issues faced on this topic.

Researchers from various institutes have contributed to the success of this Special Issue, with a total of 10 articles that were selected for publication. The content of these 10 papers covers different topics that were proposed in the text of the Special Issue. However, the topics concerning interferences with *in vitro* genotoxicity assays and proposals for nanomaterial reference controls have not been addressed as well as the approaches based on grouping, ranking, and read-across. This issue contains mostly data from *in vitro* studies. In addition to classical cell lines such as hamster and human fibroblasts [1,2] or human lung cells [3,4], new models have been also used for investigating nanomaterials genotoxicity: ToxTracker reporter cell lines [5], human amniotic cells [6] and 3D HepG2 spheroids [7]. In this issue, three papers also deal with *in vivo* studies: on the plant *Allium cepa* [8], on the tadpoles of *Xenopus laevis* [9] and on rats [10]. Moreover, a broad range of nanomaterials have been tested, including plastic particles [2] that recently became of increasing concern for environmental and public health.

Among the various assays available for investigating genotoxicity and mutagenicity, most of the papers used the comet assay to visualize the DNA fragmentation both in *in vitro* [3,4,6,7] and *in vivo* [10] studies. The micronucleus assay to detect chromosome and genome mutations was also largely performed in cell lines [2,4] as well as in whole organisms [8,9,10]. In contrast, only one article deals with the gene mutation Hprt assay [1].

Concerning the role of the physico-chemical characteristics, it was reported that the surface area is one of the dose metrics showing a better correlation with the genotoxicity of quantum dots [5], while the metal/coating agent ratio is a key parameter for the toxicity observed with the commercially available AgNPs formulation Argovit™ [8]. Similarly, the thermal reduction of graphene oxides generates a material that was no longer genotoxic at low concentrations [9]. In contrast, the type of synthesis used for producing tungstene particles did not significantly affect the toxicity [4]. The impact of size was investigated on the toxic responses either using two protocols of dispersion with TiO_2_ nanoparticles leading to different size distribution [1] or by testing similar nanomaterials with different primary sizes [5]. Such results are integrated into a “safe by design” approach by investigating solutions to decrease the genotoxicity of the nanomaterials through specific coatings, production processes or treatments as examples.

As already reported in the literature, oxidative stress was suggested as one mechanism involved in the genotoxic responses observed [2,3,4,9]. Co-exposure to nanomaterials and other compounds can affect the genotoxicity [2,6].

It has been suggested that the genotoxic effects generated by nanomaterials can be due to the dissolution and formation of ions. Nevertheless, although when ions are formed, it does not preclude that nanoforms per se can also induce some genotoxicity. In this issue, the comparison of nanoforms with an ionic counterpart has been done for aluminum after gavage to rats [10] and for silver in *Allium cepa* [8]. In both cases, the genotoxic effects observed could not be solely explained by the formation of ions from the nanoforms tested.

The testing of genotoxicity faces the tremendous number of nanomaterials that can theoretically differ for only one parameter. Therefore, high throughput methodologies can facilitate the screening. A high throughput methodology applied to the comet assay has been used in the European project NanoReg [3]. ToxTracker reporter cell lines [5] can be also a way to quickly assess the activation of cellular stress response pathways.

In conclusion, the papers presented in this Special Issue show how the evaluation of the genotoxicity remains challenging for nanomaterials. Nevertheless, this Special Issue highlights steps forward that have been investigated to overcome some of the concerns. Still, a reliable evaluation of the genotoxic hazard of nanomaterials will support the attempt to protect the health of the populations without strangling innovation.
